# The impact of brand advertising on children’s food preferences and behavioural intentions: an experimental study

**DOI:** 10.1017/S1368980025000369

**Published:** 2025-05-02

**Authors:** Christine Mulligan, Lauren Remedios, Tim Ramsay, Elise Pauzé, Mariangela Bagnato, Monique Potvin Kent

**Affiliations:** 1 School of Epidemiology and Public Health, Faculty of Medicine, University of Ottawa, Ottawa, Canada; 2 Interdisciplinary School of Health Sciences, Faculty of Health Sciences, University of Ottawa, Ottawa, Canada

**Keywords:** Brand marketing, Food and beverage brands, Children, Youth, Food marketing, Marketing restrictions, Food policy

## Abstract

**Objective:**

Despite strong evidence linking exposure to food and beverage marketing with poor diet quality and negative health impacts in children, the effect of brand marketing (i.e. marketing featuring branded content, but no food products) is uncertain. This study evaluated the impact of brand marketing *v.* product-based advertising on children’s food preferences and behavioural intentions.

**Design:**

An online survey was administered to participants randomised to one of four ad conditions; familiar product (i.e. from popular Canadian brands); familiar brand (i.e. no food product, Canadian brand); unfamiliar product (i.e. foreign products); and unfamiliar brand ad (i.e. foreign brand). Participants viewed three ads displaying features of that condition and answered three 5-point Likert-scale questions related to the study outcomes: food preference, purchase intent and pester power. The average of all outcomes determined the total impact. An ANOVA with Bonferroni *post hoc* tests evaluated differences in impact between conditions.

**Setting:**

Canada participants: *n* 1341 Canadian children (9–12 years)

**Results:**

Familiar product ads had a higher total impact on children (mean score 3·57) compared with familiar brand ads (2·88), unfamiliar brand ads (3·24) or unfamiliar product ads (3·09; *P* < 0·001 for all pairwise comparisons). Total impact was lower for familiar brand ads than for unfamiliar brand ads or unfamiliar product ads (*P* < 0·001 for all pairwise comparisons). The impact of an unfamiliar brand and product did not differ (*P* = 0·53).

**Conclusions:**

Results suggest that familiar product ads seem to have a stronger impact on children’s food preferences and behavioural intentions than familiar brand ads, unfamiliar brand ads and unfamiliar product ads.

The WHO has recommended that countries implement comprehensive policies to restrict children’s exposure to powerful food and beverage marketing^(1–3)^. This guidance stems from a robust body of literature documenting children’s vulnerability to the effects of marketing and its negative impact on children’s food preferences, purchase requests and intakes^(2,4–6)^. This problem is magnified by the fact that almost all marketing youth are exposed to promotes food and beverage products that are of poor nutritional quality and that do not align with healthy dietary guidelines^(1,2,7)^. In Canada, as in other high-income countries, children’s diets are poor and low in fruits and vegetables and high in ultra-processed foods^(8–11)^. Poor diet is a major risk factor for obesity and non-communicable disease^(12–16)^, and childhood overweight, obesity and non-communicable disease levels remain high globally, as well as in Canada^(17–22)^. Given its known impact on children’s food preferences and behaviours, food marketing has been identified as a determinant of poor diet quality in children and childhood obesity^(2,4–6,23)^.

The impact of food marketing on children is driven by both their exposure to marketing and the power (i.e. the content and design) of such marketing^(24)^. Globally, children’s exposure to food marketing is high and occurs across all media children use (e.g. television, social media, websites, mobile apps, etc.) and settings where they frequently spend time (e.g. restaurants, grocery stores, community spaces, schools, etc.)^(2,4,7,25–31)^. In Canada specifically, recent research has documented high volumes of food marketing exposure. For instance, on child-preferred websites over the course of one year, more than 54 million food ads were displayed^(32)^. Another study reported that in 2019, children (2–11 years) saw over 2000 food ads across multiple television stations during regular viewing^(33)^. Social media is also proving to be a dominant source of unhealthy food marketing exposure for children in Canada, such as through food company-created content, as well as user-generated content and influencer marketing^(28,34)^. Such volume of exposure leaves Canadian youth highly susceptible to marketing’s negative influence. However, despite ample research elucidating the impact of exposure to food marketing on children, the effects of brand marketing (i.e. marketing by food and beverage companies that is absent from any distinct food products) remain ambiguous.

Brand marketing features branding strategies, such as branded symbols or logos, that contribute to marketing power. Research has shown that this form of marketing elicits responses including brand preferences, awareness and purchasing behaviours in youth, which can drive long-term health effects and behaviours^(35)^. For example, a significant increase in brand recognition and attitudes among children was found after exposure to television and online food brand advertisements^(36)^. Other studies have shown the effectiveness of fast-food branded marketing on children’s brand awareness, food/brand preferences and recognition^(37–40)^.

Across multiple media and settings, Canadian children are exposed to brand marketing conducted by the food and beverage industry. Research has shown that on social media, brand advertisements made up 38 % of all food marketing exposures viewed by children 7–11 years old, and that on television, brand advertising is also present, though less prevalent^(28)^. While there has been some evidence documenting the extent of brand advertising being conducted by food and beverage companies in Canada, to our knowledge, there have been no studies that have directly examined the impact of brand advertising on children, especially in comparison with product-based food advertising. This is an important question as most, if not all, regulatory actions aiming to restrict food marketing to children, including the latest Canadian policy proposal (Bill C-252), have not included brand marketing within their scope^(41–44)^. Given this policy gap, additional research in this area is imperative^(37)^. As such, the objective of this study was to evaluate the impact of food and beverage brand marketing compared with product-based advertising from familiar and unfamiliar brands on children’s food preferences and behavioural intentions.

## Methods

As part of a larger study, a cross-sectional online survey was administered to 1341 Canadian children to determine the impact of food and beverage product-based ads *v*. brand ads from familiar and unfamiliar brands^(45,46)^. Participants (English or French-speaking children aged 9–12, in Canada) were recruited for this study via email by Leger, a market research company^(45,46)^. Recruitment was nationally representative, stratified by provincial population and by age/sex groups. The University of Ottawa Research Ethics Board (H-11-22-8517) approved this study.

After obtaining informed consent and assent, parents/guardians completed a short demographic questionnaire on behalf of their child, then children completed the rest of the survey. Participants were randomised to one of four conditions, for which they viewed three static ad images (e.g. not animated or video) in random order and were able to view the ad for as long as they wished before moving to the question page of the survey (which also displayed the image). The conditions were (a) familiar product ad (i.e. from brands heavily advertised in Canada), (b) familiar brand ad (i.e. no food product), (c) unfamiliar product ad (i.e. from brands not sold in Canada; control) and (d) unfamiliar brand ad (control). Two control conditions were necessary to discern between the impact of (a) a food or beverage product being present (i.e. product v. brand ad) and (b) the familiarity of the brand, with the rationale that familiarity should increase brand equity and preference, whereas unknown brands should, in theory, have little impact on children. Ad images were designed specifically for this study and were gender neutral (e.g. avoiding stereotypical gendered advertising techniques or characters) and age-appropriate. The featured brands were top brands by (either Canadian or international) market share from some of the most advertised food categories in Canada, for example, fast food, breakfast foods, candy, desserts or snacks^(25)^. A description of the images featured in each ad exposure and experimental condition is presented in online supplementary material, Supplemental Table 1.

In response to each ad exposure, participants answered three Likert-scale questions (5-points, indicated by emojis ranging from sad (1) to happy (5) faces) related to the outcome variables of interest (informed by existing literature^(4)^): (1) how much would you like to eat/drink this brand’s products (i.e. food preference); (2) would you choose to buy this brand’s products in a store (i.e. purchase intent); and (3) would you ask an adult to buy this brand’s products for you (i.e. pester power). Total impact was determined by averaging the scores from the three outcomes. It was hypothesised that familiar product ads would have the strongest impact on children, followed by familiar brand ads, unfamiliar product ads and lastly, unfamiliar brand ads.

Demographic variables were analysed descriptively. The difference in impact between conditions on the outcomes and total impact was evaluated using ANOVA models fitted with Likert scores for food preference, purchase intent, pester power and total impact as outcomes and sex (male/female), age (9–10 years/11–12 years), ethnicity (majority, minority), perceived income adequacy (inadequate/adequate) and condition as fixed factors/independent variables. Bonferroni *post hoc* tests were conducted when ANOVA yielded significant results (*P* < 0·05).

## Results

Of the *n* 1341 children who completed the survey, 49·2 % were male and 50·6 % were female, with a mean age of 10·6 years (47·4 % 9–10 years and 52·6 % 11–12 years) (Table 1).

**Table 1.** Demographic characteristics of the study sample (*n* 1341)

	*n*	%
Total sample	1341	100·0
Sex		
Female	679	50·6
Male	660	49·2
Prefer not to say	2	0·1
Age		
11–12 years	706	52·6
9–10 years	635	47·4
	Mean	sd
Age	10·6	1·1) years
	*n*	%
Ethnicity*		
Majority	869	64·8
Minority	457	34·1
Did not answer	15	1·1
Perceived income adequacy^†^		
Adequate	804	60·0
Inadequate	530	39·5
Did not answer	7	0·5
Province/region of residence		
West (BC, AB)	323	22·6
Prairies (SK, MB)	91	6·4
Ontario	523	36·5
Quebec	318	22·2
East (NL, NS, NB, PE)	85	5·9
North (YT, NT, NU)	1	0·1

*Ethnicity was categorised as ‘majority’ (i.e. only ‘White (European descent)’ was selected) and ‘minority’ (i.e. any other ethnicity group(s) were selected, including when in addition to ‘White (European descent)’ being selected). ^†^Perceived income adequacy was categorised as ‘adequate’ (responses of either very easy, easy and neither easy nor difficult when asked how difficult or easy it is for you to make ends meet?) or ‘inadequate’ (responses of difficult or very difficult).

**Figure 1:**
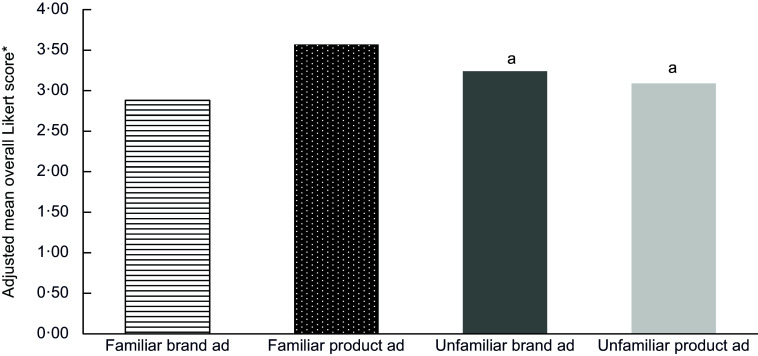
Total impact of brand ads *v*. product-based ads on children’s (*n* 1341) food preferences and behavioural intentions.

Average total impact significantly differed across almost all product and brand ad types (Figure 1). Total impact was significantly higher from exposure to familiar product ads (mean score 3·57) compared with familiar brand ads (2·88), unfamiliar brand ads (3·24) or unfamiliar product ads (3·09; *P* < 0·001 for all pairwise comparisons). Exposure to familiar brand ads resulted in significantly lower total impact than unfamiliar brand ads or unfamiliar product ads (*P* < 0·001 for all pairwise comparisons). There was no difference in total impact from exposure to unfamiliar brand ads or product ads (*P* = 0·53).

All response outcomes significantly differed by condition (Table 2). The effect of exposure to familiar food product ads on preference (mean score 3·59) was significantly greater compared with all other conditions (*P* < 0·001) while the effect of exposure to unfamiliar brand or food ads on preference did not significantly differ (mean scores 3·28 and 3·12, respectively; *P* = 0·31). Likewise, average purchase and pester responses were greater following exposure to familiar product ads compared with all other conditions (*P* < 0·001 for all pairwise comparisons); however, purchase and pester did not differ between the unfamiliar brand and product ad conditions (*P* = 0·99 and *P* = 0·67, respectively).

**Table 2.** Total impact and impact of brand ads *v*. product-based ads on children’s (*n* 1341) food preference, purchase intent and pester power

Experimental condition:	Familiar brand ad	Familiar product ad	Unfamiliar brand ad		Unfamiliar product ad
	Adjusted mean	Adjusted mean	Adjusted mean	Adjusted mean	*P* value
Food preference					
Overall	2·80	3·59	3·28^a^	3·12^a^	*P* < 0·001
Sex					0·25
Male	2·94	3·57	3·27	3·19	
Female	2·66	3·62	3·29	3·06	
Age					0·4
9–10 years	2·91	3·6	3·25	3·19	
11–12 years	2·7	3·59	3·31	3·06	
Ethnicity					0·54
Minority	2·94	3·69	3·38	3·14	
Majority	2·67	3·5	3·18	3·11	
Perceived income adequacy					0·22
Inadequate	2·88	3·58	3·2	3·18	
Adequate	2·72	3·61	3·36	3·07	
Purchase intent					
	Adjusted mean	Adjusted mean	Adjusted mean	Adjusted mean	*P* value
Overall	2·89	3·53	3·21^a^	3·07^a^	*P* < 0·001
Sex					0·39
Male	3·03	3·52	3·23	3·14	
Female	2·74	3·54	3·19	3·01	
Age					0·49
9–10 years	2·98	3·53	3·22	3·17	
11–12 years	2·79	3·54	3·2	2·98	
Ethnicity					0·54
Minority	3·01	3·62	3·32	3·09	
Majority	2·76	3·45	3·1	3·06	
Perceived income adequacy					0·28
Inadequate	2·98	3·51	3·14	3·1	
Adequate	2·79	3·56	3·28	3·05	
Pester power					
	Adjusted mean	Adjusted mean	Adjusted mean	Adjusted mean	*P* value
Overall	2·95	3·57	3·23^a^	3·08^a^	*P* < 0·001
Sex					0·61
Male	3·04	3·58	3·21	3·15	
Female	2·86	3·56	3·24	3·01	
Age					0·71
9–10 years	3·05	3·59	3·23	3·14	
11–12 years	2·85	3·54	3·23	3·01	
Ethnicity					0·55
Minority	3·09	3·65	3·34	3·09	
Majority	2·81	3·48	3·12	3·06	
Perceived income adequacy					0·29
Inadequate	3·08	3·57	3·17	3·09	
Adequate	2·82	3·57	3·29	3·06	
Total impact					
	Adjusted mean	Adjusted mean	Adjusted mean	Adjusted mean	*P* value
Overall	2·88	3·57	3·24^a^	3·09^a^	*P* < 0·001
Sex					0·41
Male	3	3·56	3·24	3·16	
Female	2·76	3·57	3·24	3·03	
Age					0·52
9–10 years	2·98	3·57	3·23	3·17	
11–12 years	2·78	3·56	3·25	3·02	
Ethnicity					0·51
Minority	3·01	3·66	3·34	3·11	
Majority	2·75	3·47	3·14	3·08	
Perceived income adequacy					0·25
Inadequate	2·98	3·55	3·17	3·13	
Adequate	2·78	3·58	3·31	3·06	

*Adjusted means based on ANOVA models fitted with Likert scores for food preference, purchase intent, pester power and total impact as outcomes; sex (male/female), age (9–10 years/11–12 years), ethnicity (majority, minority), perceived income adequacy (inadequate/adequate) and experimental condition as fixed factors/independent variables. Within rows, means that share subscript letters have means that do not differ by *P* < 0·05 according to Bonferroni multiple comparisons. ^†^*P* values < 0·05 were considered to be statistically significant. ^‡^Ethnicity was categorised as ‘majority’ (i.e. only ‘White (European descent)’ was selected) and ‘minority’ (i.e. any other ethnicity group(s) were selected, including when in addition to ‘White (European descent)’ being selected). ^§^Perceived income adequacy was categorised as ‘adequate’ (responses of either very easy, easy and neither easy nor difficult when asked how difficult or easy it is for you to make ends meet?) or ‘inadequate’ (responses of difficult or very difficult).

## Discussion

This study aimed to elucidate the impact of brand marketing compared with product-based marketing on children’s food preferences and behavioural intentions. Results showed that ads featuring familiar food products had a stronger total impact on children than familiar brand ads and unfamiliar brand and product ads. This trend was consistent across all other study outcomes (i.e. food preference, purchase intent and pester power).

These results suggest that for familiar brands, the presence of a food product may in itself be a powerful aspect of food marketing, aligning with the theory that marketing is a combination of the ‘4Ps’: price, promotion, placement and in this case, product^(47,48)^. Findings also suggest that familiarity is a contributing factor to the impact of food marketing on children, since ads for familiar products were found to be more impactful than those for unfamiliar products, and there was no difference in impact between unfamiliar brand and product ads. It would be reasonable to expect that familiar brand ads would also have a stronger impact on participants than unfamiliar brand ads (as hypothesised); however, this was not observed in our study. In fact, not only did unfamiliar brand ads elicit higher scores than familiar brand ads, but familiar brands also elicited average scores that could be considered low if not somewhat negative (i.e. below 3 on the Likert scale). This unexpected result may be due to other child-appealing aspects of the ad exposures in the familiar brand ad condition (e.g. colour, other design elements^(49)^) that were not consistent across conditions and may not have been perceived as positively by participants or could have simply been an anomalous result.

It was interesting that the unfamiliar brand and product ad conditions had a similar impact on children, given that Canadian children have likely never been exposed to ads for the foreign brands that were displayed and, therefore, probably did not have existing preferences or loyalty towards these brands. Consequently, it could have been reasonable to assume that the unfamiliar food ads would have had a stronger impact than unfamiliar brand ads, given the presence of a food product, *v*. just an image promoting an unknown brand. Again, these results could be explained by other design elements of the unfamiliar brand ads that were appealing to children, despite the absence of a food product.

While the familiar brand ad condition in this study was not found to be particularly impactful, brand marketing remains an important tactic used by food and beverage companies to build brand equity and loyalty^(35–39)^. Importantly, the familiar product ad condition in this experiment, which combined aspects of familiarity and the presence of a food product, was found to have the strongest impact on children’s desire to consume, purchase and pester about those products. How children’s exposure to brand marketing contributes to building this familiarity and potentially increasing the impact of product-based marketing is worth examining.

This research was strengthened by using a large and nationally representative sample of Canadian children. Efforts were made to reduce bias due to pre-existing preferences or random error by using multiple ad exposures per experimental condition and designing the survey images such that they were gender neutral and from a variety of food categories. Randomisation was also employed in assigning conditions and determining the order of ad exposures within the condition. A strong analytic approach (ANOVA) allowed the comparison of outcomes between experimental conditions and to adjust for sociodemographic variables. *Post hoc* Bonferroni tests deepened our analysis through the identification of significant pairwise comparisons. However, the generalisability of our findings may be limited, as the study sample consisted mainly of participants identifying as White and of higher income, a trend often seen with online survey panels^(50)^. Children were also only exposed to three images intended to be universally appealing to reduce fatigue, increase feasibility and limit bias. However, children have individualised preferences that may not have been fulfilled by the experimental ads, potentially reducing the variability or strength of the response outcomes. Due to poor quality self-reporting of participants’ height and weight, BMI or weight status was not able to be included in this analysis as a potential covariate on the outcomes^(45,46)^.

While gaps remain in our understanding of how children respond to the brand advertising conducted by the food and beverage industry, this study suggests that familiarity matters and that the presence of a food product can generate power and contribute to the marketing’s overall impact on children. Given the volume of brand advertising that children are exposed to, primarily for unhealthy food categories^(28)^, further examination into what thoughts, ideas or food-related associations are triggered by brand ads is warranted, particularly as countries like Canada are considering excluding brand marketing from marketing regulations^(41)^. Increasing our understanding of brand marketing is critical to informing the development of comprehensive marketing policy in Canada and globally.

## Supporting information

Mulligan et al. supplementary material Mulligan et al. supplementary material

## Supplementary material

For supplementary material accompanying this paper, visit https://doi.org/10.1017/S1368980025000369.
