# Supplementary material for: The impact of brand advertising on children’s food preferences and behavioural intentions: an experimental study
Source: Public Health Nutr. 2025 May 2;28(1):e90. doi: 10.1017/S1368980025000369 (PMC12100560; doi:10.1017/S1368980025000369)
Supplement: Mulligan et al. supplementary material [file S1368980025000369sup001.docx]

| **Experimental condition** | **Ad Exposure** | **Brand (de-identified)** | **Product category** | **Ad features** |
| --- | --- | --- | --- | --- |
| 1. Familiar product ad (i.e., from brands heavily advertised in Canada) | 1 | A | Snack | Brand logo and children’s toy visible; food product visible |
|  | 2 | B | Fast Food | Brand logo and children’s toy visible; food product visible |
|  | 3 | C | Fast Food | Brand logo and brand character visible; food product visible |
| 2. Familiar brand ad (i.e., no food product) | 1 | A | Snack | Brand logo visible; food product removed |
|  | 2 | B | Fast Food | Brand logo visible; food product removed |
|  | 3 | C | Fast Food | Brand logo and branded character visible; no food product visible |
| 3. Unfamiliar product ad (i.e., from brands not sold in Canada; control) | 1 | D | Yogurt | Brand logo, name, character and child visible; food product visible |
|  | 2 | E | Cereal | Brand logo, name, character and child visible; food product visible |
|  | 3 | F | Fast Food | Brand logo visible; food product visible |
| 4. Unfamiliar brand ad (control) | 1 | D | Yogurt | Brand logo, name, character and child visible; no food product visible |
|  | 2 | E | Cereal | Brand logo, name, character and child visible; no food product visible |
|  | 3 | F | Fast Food | Brand logo visible; no food product visible |

**Supplementary Table 1**. Description of images used in each experimental condition and ad exposure
